# The Awake Craniotomy: A Patient’s Experience and A Literature Review

**DOI:** 10.7759/cureus.26441

**Published:** 2022-06-29

**Authors:** Tye Patchana, Jose A Lopez, Gohar Majeed, Alison Ho, Tony Alarcon, Natasha Plantak, Peter Vu, Javed Siddiqi

**Affiliations:** 1 Department of Neurosurgery, Riverside University Health System Medical Center, Moreno Valley, USA; 2 Medicine, St. George's University School of Medicine, West Indies, GRD; 3 Department of Neurosurgery, Desert Regional Medical Center, Palm Springs, USA; 4 Internal Medicine, University of Nevada Las Vegas, Las Vegas, USA; 5 Department of Neurological Surgery, Arrowhead Regional Medical Center, Colton, USA; 6 Department of Pharmacy, Arrowhead Regional Medical Center, Colton, USA; 7 Department of Neurosurgery, Arrowhead Regional Medical Center, Colton, USA; 8 Department of Neurosurgery, California University of Science and Medicine, Colton, USA

**Keywords:** craniotomy while awake, patient psychology, awake brain surgery, patient experience, awake craniotomy

## Abstract

We present a case report and a literature review of the awake craniotomy procedure for mass resection, with emphasis on the historical aspects, anatomical and surgical considerations, and, uniquely, a patient’s experience undergoing this procedure. This procedure is a safe and effective method for lesion resection when working in and around eloquent brain. We have described our process of guiding a patient through an awake craniotomy procedure and detailed the patient's experience in this study. We also conducted a systematic literature review of studies involving awake craniotomy over three years, 2018-2021. Lastly, we compared the methodology used by our institution and the current mostly used methods within the neurosurgical community. Several studies were identified using PubMed and Google Scholar. Awake craniotomy is a safe and effective method of achieving a high rate of resection of lesions located in and around the eloquent cortex with a low degree of postoperative neurological deficit.

## Introduction

No surgery is as famous as awake craniotomy, which we will explore both from a literary review and from a patient’s direct experience. Many previous studies have demonstrated that the extent of resection (both of high- and low-grade gliomas) portend increased survival rates in patients. Thus, when lesions are located within the eloquent cortex, awake craniotomy has been shown to be the gold standard [1]. The term “eloquent” was first used in the context of brain anatomy in a 1951 paper, which described the surgical management of brain abscesses [2].

This procedure is associated with a greater extent of resection and a lower incidence of postoperative neurological deficits [3]. Initially associated with surgical treatment for intractable epilepsy, this method has since been increasingly utilized for glioma resection [4]. This procedure can be a frightening experience for patients who are fraught with preoperative anxiety related to the fact that surgery is being performed on the very organ that makes us unique humans. In the patient’s mind, this naturally brings many different thoughts, such as: Will I be the same person I once was following my surgery? Will I be able to perform my familial duties and continue working to support my family? Will there be significant pain and discomfort during the procedure? The field of neurosurgery has progressed since our evidence of the very first craniotomies in Aztec times. There is evidence that those who underwent such procedures in the distant past survived; however, it must have been a very unpleasant experience for those patients. With the advent of anesthesia and antibacterial agents, this procedure is much safer today [5].

Awake craniotomy is utilized in the resection of lesions located in proximity or within the eloquent brain cortex and finds the most utility in both epilepsy surgery and tumor resection. The procedure allows for intraoperative, real-time identification of eloquent areas to avoid compromise of the patient’s neurological function. Two anesthetic methods used to complete the procedure include monitored anesthesia care (MAC) and asleep-awake-asleep techniques. Though the former has been found to be associated with a lower risk of surgical failure and shorter procedure time, MAC is also the authors’ preferred method of utilizing anesthesia during the procedure [6]. Further, a comparison of glioma resection between craniotomy under general anesthesia and awake craniotomy has also shown several benefits for the performance of craniotomy while awake [7]. The procedure revolves around appropriate analgesia, typically achieved with neurosurgical expertise in scalp block and the use of appropriate sedation and analgesia during the procedure. Dexmedetomidine (an alternative to propofol) has previously demonstrated a high degree of safety as well as efficacy in previous studies [4]. Indeed, several case series have been presented over the past decade describing the use of dexmedetomidine in the clipping of aneurysms, resection of tumors associated with the optic radiations, and even in deaf patients who communicated using sign language [8-10].

Appropriate patient selection is paramount in assessing who will benefit from an awake craniotomy procedure. The patient’s ability to participate and cooperate with commands is a prerequisite to a successful operation. Contraindications to surgery include mental confusion, poor compliance, and inability to concentrate [11]. At least one study has also shown that fear of pain was positively correlated with pain during the procedure, which was revealed through psychological questionnaires [12]. It is known that patients with brain tumors have higher levels of mental distress, anxiety, depression, and elevated stress in the days following intervention when compared to those with tumors from other body sites [13,14]. Interestingly, prior studies have shown mixed results regarding awake craniotomy with some studies showing an association with negative psychological sequelae such as increased arousal, fear, and avoidance of stimuli [15]. Others have demonstrated postoperative negative psychological phenomena that did not deviate from the preoperative baseline [16].

Although comprehensive reviews have been written on the technique of awake craniotomy, this study will focus on both the authors’ and, more importantly, the patient’s experience of awake craniotomy at a single institution in the United States.

## Case presentation

We will discuss a single patient’s comprehensive experience from discussing his planned procedure with his family, the pre-procedural anxiety, the preoperative scalp block process, and his experience during the procedure in this section.

Our patient is a 50-year-old right-handed male who presented with a first-time complex seizure and episode of emesis. He had also endorsed a month-long history of intermittent expressive aphasia and personality changes. The patient received a computed tomography (CT) of his head at an outside facility demonstrating an 18 mm left frontal ring-enhancing lesion with associated vasogenic edema and was transferred to our facility for evaluation by our neurosurgical service. He reported a benign medical history, though endorsed a maternal family history of pheochromocytoma, and a deceased brother secondary to an unknown brain lesion. The patient was neurologically intact on examination, receiving high-dose steroids prior to his evaluation by our neurosurgical service. The patient had an unremarkable chest, abdomen, and pelvis CT with IV contrast and computed tomography angiogram (CTA) of his head and neck. Following admission, several discussions took place between the patient and our neurosurgical service. We believed due to the proximity of the pars triangularis of the inferior frontal gyrus (associated with Broca’s area), an awake craniotomy operation would be the best neurosurgical option for the patient.

Pre-procedural scalp block

**Figure 1:**
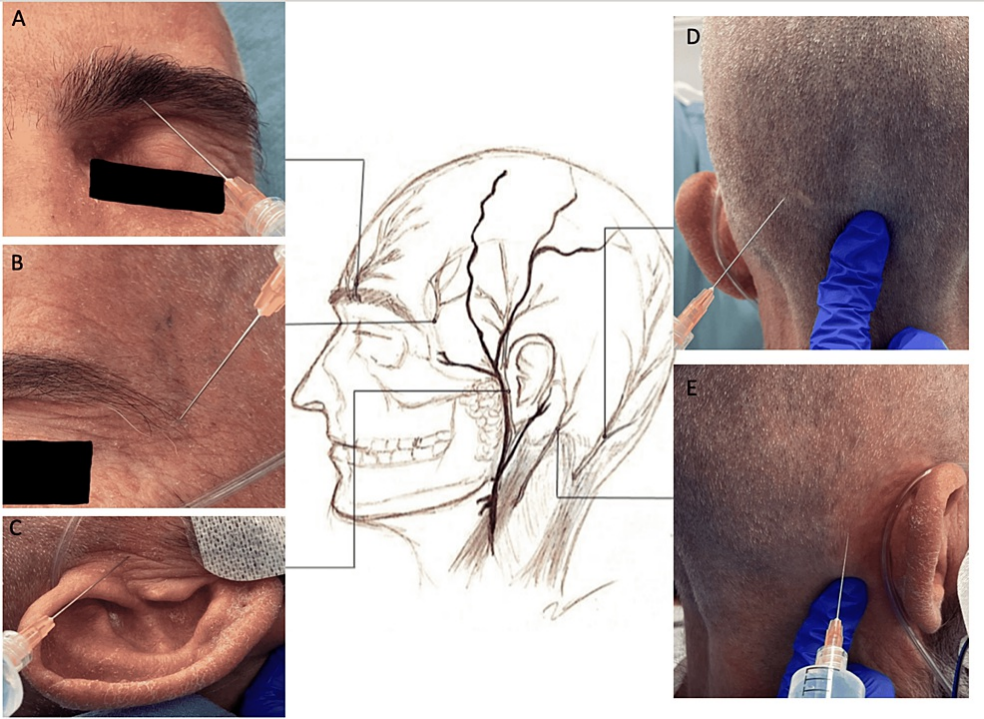
Regional scalp block for awake craniotomy: (a) supraorbital nerve, (b) zygomaticotemporal nerve, (c) auriculotemporal nerve, (d) greater occipital nerve, and (e) lesser occipital nerve. Image credit: Alison Ho.

Proper analgesia is imperative to the patient’s experience during awake craniotomy. Sensory innervation to the face and scalp is supplied by several important branches that must be properly anesthetized prior to the craniotomy. Important innervation is supplied by the supraorbital and supratrochlear nerves above the orbit (Figure 1, Panel A), zygomaticotemporal and auriculotemporal nerves (Figure 1, Panels B and C) more laterally, and the greater occipital and lesser occipital nerves (Figure 1, Panel D) posteriorly. It is important to consider bilateral nerve blockade if the incision is to cross the midline to achieve appropriate analgesia. This is especially important in the setting of Mayfield pins used to hold the head during surgery. However, the authors’ preference has been to forgo Mayfield pinning, which has not had any deleterious effects on surgery and increases patient comfort and anxiety dramatically. Furthermore, the authors’ approach to scalp block utilizes three medications - Lidocaine ointment 5%, Bupivacaine 0.5% with epinephrine 1:200,000, and Lidocaine 0.5% with epinephrine 1:200,000.

Awake craniotomy procedure

The patient was brought to the preoperative area prior to surgery and given 1 mg sublingual Ativan for anxiety. The hair was clipped around the planned incision site, and areas of nerve block were marked and prepped. Topical 5% Lidocaine was applied to the scalp sites prior to performing nerve blocks. We preferred a mixture of 0.5% Lidocaine with 1:200,000 epinephrine and 1% Bupivacaine with 1:200,000 epinephrine. This solution was used to perform blocks at the following sites: supraorbital/supratrochlear nerve, zygomaticotemporal nerve, auriculotemporal nerve, lesser occipital nerve, and greater occipital nerve. Ultrasound was used to identify the superficial temporal artery and the occipital artery, thus allowing us to avoid their incorporation during the scalp blocks within the temporal and occipital areas. The patient rested comfortably in the preoperative area for approximately two hours prior to being taken to the operating room (OR).

The patient was brought into the OR, and a standard time-out was performed. The patient was positioned on the OR table with the horseshoe head holder and left side up. All pressure points were carefully padded. A warm blanket was placed on top of the patient to keep him comfortable. The patient's head was gently turned to expose the left frontotemporal region and the planned incision. Signs were posted in the OR stating "Discretion, patient awake." The incision line was injected with a local anesthetic of 1% Lidocaine with epinephrine. A sterile sharp needle was used to test the effectiveness of the regional nerve block and incisional local anesthetic. The patient was noted to have no pain subjectively or any change in vital signs indicative of pain or discomfort. Next, we registered the intracranial stealth navigation system (Stealth®, Medtronic, Dublin, Ireland). Vital structures including the tumor borders were identified and mapped out. The planned craniotomy along with the trajectory for entry and target was then outlined. The incision was re-marked. A final time-out was held with nursing staff, anesthesia, and neurosurgery to confirm the correct patient, the correct site of surgery, the correct procedure, the proper positioning, padding of the patient, and line access as agreed upon by nursing and anesthesia staff. A 10/10 drape was placed on the forehead of the patient.

The patient was told that he would be informed prior to every step, so there would not be any surprises for him. Frequent checks occurred to ensure that the patient was comfortable and free of anxiety. Anesthesia stood ready to temporarily sedate the patient for parts of the surgery, at our request. Next, the patient was prepped and draped in a standard sterile fashion. A member of the team was making constant communication with the patient under the drapes but away from the sterile field. A standard bone flap was turned to expose the dura in the desired operative corridor. A tuberculin syringe was then used to inject 0.5% Lidocaine with epinephrine between the two layers of the dura, parallel to the middle meningeal artery and branches. An ultrasound probe was then used to confirm the location of the mass prior to making the dural opening. The dura was then opened in a cruciate fashion using a #11 blade and dural scissors. The leaflets were reflected away. A strip electrode was then placed in the subdural space posteriorly to perform direct cortical electroencephalogram (EEG) monitoring; two electrodes were also placed by the neurophysiology tech in the patient's face and hands to monitor seizure-associated electromyography (EMG) changes.

The location of the mass was visualized with ultrasound to plan the corticectomy. Anesthesia staff were then asked to make the patient more awake by weaning down the IV anesthetics. A team member was engaged in direct communication with the patient, and he was asked to perform simple tasks including sticking his tongue out, counting backward from 20, and doing simple math. Cortical stimulation was performed using a probe for language area mapping. No speech arrest, paraphasia, or hesitations were noted. We determined that the area of planned corticectomy did not stimulate any functional significance. The surgical microscope was brought into the surgical field, and the mass was resected in a standard microneurosurgical fashion. Following the procedure, the patient’s speech and motor exam were again tested, and no new deficits were appreciated. He was transferred back to an ICU bed. The patient was able to have a full conversation with the neurosurgery team after the drapes were taken off. Pupils were noted to be 3-2 mm briskly reactive bilaterally. The patient’s wife was updated in real-time in the OR by the attending neurosurgeon and the neurosurgery team; the patient was also able to speak with his wife from the OR. Postoperative care proceeded without any complications.

Post-procedural patient experience

Our patient successfully underwent an awake craniotomy procedure for mass resection without any neurological deficit or complications. The patient was interviewed on postoperative day number one by our neurosurgical team to determine the patient's experience after undergoing awake craniotomy. We sought to examine the patient’s level of anxiety and discomfort, both psychologically and physically, throughout the periprocedural period and postoperatively. The following section demonstrates our questions and the patient’s responses.

What was it like to consider the option of having an awake craniotomy procedure?

The patient expressed that he was pleased with his decision to go forward with an awake procedure as opposed to the alternative of surgery under general anesthesia. He expressed surprise and gratitude at the level of attention paid to him throughout the procedure from the anesthesia, neurosurgical, neuromonitoring, and OR teams. He was very aware throughout the process that a large amount of personnel and equipment were arranged in such a way as to give him the best outcome.

What was the process like to discuss with family?

The patient endorsed that his family was worried and did have some reservations regarding the procedure. They expressed concern surrounding the notion of whether the patient would still be himself following surgery. They wondered how extensive the recovery process would be and how long it would take. The patient and his family worried about continued seizures especially in the setting of the patient’s occupation as a truck driver.

What was the process of the scalp block like and the ensuing surgical procedure once in the operating room?

Prior to the procedure, we discussed with the patient that previous patients had reported the sounds experienced during the procedure were the most unsettling aspect. The patient corroborated this. During the initial scalp block injections and throughout the craniotomy, the patient stated that he could hear the anesthetic being injected into his scalp and described this sound as “ice cracking.” Additionally, the worst part was the initial scalp block injections, which felt to be cold. Once the patient entered the OR, he endorsed being able to hear all conversations around him. The procedure appeared to proceed quickly to the patient. He felt the addition of a person by his side, talking to him throughout the procedure, made him feel not alone. The patient felt that this step in the procedure was important to him. He also always felt that he knew what was happening due to the constant communication from the neurosurgeons as well as the reassurance that things were proceeding according to plan. He felt that the procedure would have been difficult if he had to stare at a wall, wondering about the progress of the procedure.

## Discussion

A variety of authors used different methods to measure patients’ psychological experiences while undergoing awake craniotomy. These include the use of interpretative phenomenological analysis (IPA) to explore how people made sense of the awake craniotomy experience [17], use of qualitative descriptive surveys [18], state-trait anxiety inventory (STAI, self-reported inventory established by Spielberger, with a higher score corresponding to higher anxiety), and Mann-Whitney U test (to determine the effect of music toward anxiety level and physiological responses) [19].

Attempts have been made to explain the participants trying to make sense of their experience - also known as "double hermeneutic" in phenomenological studies [20]. Zemmoura et al. used three standardized questionnaires: the Cohen perceived stress scale, the posttraumatic stress disorder checklist scale, the peritraumatic dissociative experience questionnaire, and a fourth questionnaire designed specifically for their study [21]. Hejrati et al. used a numerical rating scale (NRS) for preoperative assessment of fear and pain, a hospital anxiety and depression scale (HADS-D) (German version) to screen for fear and depression in patients with physical diseases or symptoms, a patient health questionnaire (PHQ-D) for posttraumatic stress disorder (PTSD)-related symptoms, and brief pain inventory (BPI) to measure pain and its interference with seven daily activities [15].

The psychology and patient’s experience are best summarized by the three superordinate themes: use of self-preservation strategies prior to and during awake craniotomy, a bizarre yet pleasant operation experience, and the need for more concrete information prior to surgery. Each of the three superordinate themes appeared to be embedded in a core theme of having a good relationship with the neurosurgeon [17].

Even though previous studies reported awake craniotomy is well tolerated and has great patient satisfaction, anxiety had been reported as the most common psychological phenomenon [15]. Anxiety is one example where patients use different self-preservation strategies to cope with the experience of preparing for the surgery by making a conscious effort to not think about the event. Some of the coping mechanisms patients used were avoidance, distraction, external focus, and humor [17]. Creating a “safe place” may be a form of self-preservation. The anesthesiologist helped patients construct this safe place (imaginary place) through a short hypnosis session to feel safe and protected from the mental stress or anxiety from the awake craniotomy [22]. Patients incorporate visual, hearing, smell, and taste to create these safe or pleasant places during multiple sessions in the hypnosis clinic with the goal of recreating a relaxing environment during both the induction and awakening phase of the operation [22].

Similarly, an interesting case study of an 11-year-old female patient with a tumor in her right motor cortex (presumed to be a dysembryoplastic neuroepithelial tumor [DNET]) had a simulated surgical experience by replicating a theatrical setting in which the patient dressed in a theater attire, brought into the OR in a trolley, transferred onto the OR bed, and placed in the exact position as the actual surgery [23]. The theatric experience created a safe place for the patient to be psychologically and emotionally prepared for the awake craniotomy. Music demonstrated a significant benefit in the self-preservation of coping with the awake craniotomy procedure. Potters and Klimek believed music acted as a distraction from the actual surgical procedure [24]. Wu et al. showed music had a significant difference with lower anxiety levels and physiological responses such as lower systolic pressure, diastolic blood pressure, and heart rate in the experimental group than in the control group but showed no statistical difference in the respiratory rate between the two groups [19]. Potters and Klimek also believe the applications of music provide lower anxiety scores, higher postoperative patient satisfaction, and improved sedation of patients [24].

Prior to the awake craniotomy, the patient's expectations of the procedure compared to the actual event are not coordinated resulting in the feeling of having a weird or bizarre experience perioperatively. Bajunaid and Ajlan found that most of their patients had auditory recollections from the operation and no pain perception, and none of the patients reported that awake craniotomy was more difficult than anticipated [18]. However, other patients may have a negative bizarre experience or a negative consequence on their mental health due to the traumatic incidence of the awake craniotomy. Zemmoura et al. used hypnosis to improve comfort during surgery and improve the postoperative quality of life by avoiding the potentially traumatic experience of the awake craniotomy [21]. Due to some patients finding the awake craniotomy positively or negatively bizarre, for the patient to control the emotion felt during the procedure, they rely on their self-preservation strategies and occupied their minds by conversing with the surgical team [17].

Anesthesiologists play a crucial role in the patient's experience of awake craniotomy. Ma and Uejima's case study of a 32-year-old male with a past medical history of PTSD involved a neuroanesthesiologist preoperatively for an awake craniotomy in preparation for each step of the operation. A focus was placed on when to ask questions to get a sense of control and to be able to communicate during symptomatic distress [25]. Children undergoing awake craniotomy met with the anesthesia team for hypnosis conditioning (three weeks and one week before surgery) to be used during anesthetic induction, preoperative awakening, and periods without stimulation [22]. Intraoperatively, the anesthesiologist assessed the effectiveness of hypnosis by using objective trance symptoms (eye-roll sign, tingle) and adapted the storytelling they created preoperatively based on the patient’s habits and hobbies [21]. Potters and Klimek added negative suggestions by the anesthesiologist that could lead to the nocebo effect and reframed it into positive suggestions, which show decreased pain, anxiety, and the use of analgesics [24].

The need for more concrete information prior to surgery was presented by the patients due to the inability to relay information back to family and friends and understand the operation procedures. However, despite the dissatisfaction, it was hypothesized that failure to seek out information was due to patients utilizing their relationship with the neurosurgeon to compensate for the lack of concrete information [17]. Potters and Klimek showed that giving detailed explanations with proper counseling, shaping perceptions, and managing expectations before the operation alleviate anxiety and prevent acute psychological stress reaction or acute withdrawal of the consent of the patient before or during the procedure [24]. Bajunaid and Ajlan's cohort of patients reported less anxiety and tolerated the experience from the OR to the recovery phase due to the detailed explanation, preoperatively, regarding the routine of the operation that occurs before, during, and after the surgery [18]. Ma and Uejima's PTSD patient was given a detailed explanation prior to the awake craniotomy, especially recognizing potential triggers such as loud noise during the operation (advocating for surgeons to warn patients before using the drill and electrical cautery) [25]. The approach toward children may be different. Riquin et al. had pediatric patients undergoing awake craniotomy meet with another child that had the operation, visited the OR to meet the surgical and anesthetic team, and showed pictures and a video describing the atmosphere of the OR [22].

Fletcher et al. believed that patients utilized their self-preservation (based on their relationship with the neurosurgeon) by entrusting the neurosurgeon of giving the responsibility to decide whether to go ahead with the procedure and avoid facing the implications of the decision themselves [17]. An extension of the doctor-patient relationship to other members of the surgical team beyond the neurosurgeon, such as the relationship between the anesthesiologist carrying out the procedure, is mandatory for a successful awake craniotomy making the personal relationship one of the most essential aspects of a premedication visit [25]. However, without the combination of having a good relationship with the neurosurgeon and self-preservation, patients would have focused on the more bizarre aspect of the operation and potentially suffered increased apprehension levels [17]. Regarding PTSD patients who qualified for awake craniotomy, a way to decrease the apprehensiveness or bizarre aspect of the operation will be a multidisciplinary approach with a thorough preoperative neuropsychological assessment and counseling [25].

A traumatic experience during an awake craniotomy is prejudicial to the outcome of the quality of life [21]. Riquin et al. had a child psychiatrist evaluate the traumatic impact of awake craniotomy understanding and revealed the exposure is linked to elevated levels of intrusion, anxiety, and intense imagination about the surgical sequence [22]. There were also concerns of psychosomatic strain in susceptible patients with psychiatry history with both short- and long-term psychological sequelae [25]. It is important to have good management perioperatively to give the best quality of life by decreasing the potentially traumatic experience of the awake craniotomy. Preoperative mental and psychological preparation has a direct impact on the psychological experience, which subsequently favors a positive neuropsychological outcome [15,23,26]. For a successful awake craniotomy, Girvin stated “the psychological preparedness of the patient is the most important consideration” [27]. Labuschagne et al. noted that 10%-15% of adult patients will report severe anxiety during the awake craniotomy [23]. Parents of children undergoing awake craniotomy experience their own psychological distress. Parents are assessed to see if they have the capacity to support their child and noted that the risk of the child developing PTSD depends on the seriousness of their event, the age of the child, and the parental distress. For that reason, parents are their “major protective factor” and their suffering from the event can become "agents of traumatic experience” [22]. Hejrati et al. findings noted that the awake craniotomy did not induce any shift in the medical level of anxiety, depression, and stress symptoms, inferring the operation did not add the systemic change in the psychological symptoms at the individual level. However, they found anxiety and depression were moderate to strongly associate over time, fear and pain were related intraoperatively, preoperative fear and anxiety were related to postoperative pain intensity, and preoperative fear and anxiety were related to postoperative pain interference [16].

More studies are needed to understand the psychology of the patient’s experience during awake craniotomy. Not only is the procedure safe and effective, but we also must consider the patient’s psychological aspect to mitigate the mental stress before, during, and after the procedure. Studies so far demonstrated that a good relationship between the patient and the neurosurgeon/surgical team is crucial to the patient’s experience [17,24]. Multiple factors such as self-preservation, music, trust, and being informed about the surgery are needed preoperatively to improve the psychological outcomes leading to, during, and after awake craniotomy [17-19]. Music interventions are easily assessable and convenient to make the overall awake craniotomy experience tolerable. Music helps reduce the secretion of catecholamines that regulate autonomic functions and improve a patient’s respiratory rate, heart rate, blood pressure, body temperature, and muscle tension [28]. Hypnosis conditioning may be effective and easily performed in creating a spatiotemporal distortion [22]. It may be considered a suitable alternative for older patients including the two limitations (management of airways or long walking period) of the asleep-awake-asleep method (still the gold standard) of the awake craniotomy [21]. Labuschagne et al. believed employing a theatric experience as closely as possible to the actual surgical event is an important consideration to help with the psychological anxiety and potential sequelae of the awake craniotomy [23]. We believe the overall patient psychological experience plus the relationship with the neurosurgeon/surgical team are crucial throughout the entire process from the initial consideration of the awake craniotomy to the postoperative recovery.

Limitation

This review included a limited number of studies. Additionally, our study only included one patient that relied on that patient’s subjective experience. Objective data/questionnaires were not included in our study.

## Conclusions

Awake craniotomy is a safe and effective method for achieving a high rate of resection of lesions located in and around the eloquent cortex with a low degree of postoperative neurological deficit. A review of this neurosurgical team’s experience is highlighted with a focus on both the authors and, more importantly, the patient’s experience of awake craniotomy at a single institution in the United States.
